# The unique structural features of carbonmonoxy hemoglobin from the sub-Antarctic fish *Eleginops maclovinus*

**DOI:** 10.1038/s41598-019-55331-3

**Published:** 2019-12-12

**Authors:** Nicole Balasco, Luigi Vitagliano, Antonello Merlino, Cinzia Verde, Lelio Mazzarella, Alessandro Vergara

**Affiliations:** 1 Institute of Biostructures and Bioimaging, CNR, Via Mezzocannone 16, Naples, Italy; 2 Dept. Chemical Sciences, University of Napoli “Federico II”, Via Cinthia, 80126 Naples, Italy; 3 Institute of Biosciences and BioResources, CNR, Via Pietro Castellino 111, 80131 Naples, Italy

**Keywords:** X-ray crystallography, Proteins

## Abstract

Tetrameric hemoglobins (Hbs) are prototypical systems for the investigations of fundamental properties of proteins. Although the structure of these proteins has been known for nearly sixty years, there are many aspects related to their function/structure that are still obscure. Here, we report the crystal structure of a carbonmonoxy form of the Hb isolated from the sub-Antarctic notothenioid fish *Eleginops maclovinus* characterised by either rare or unique features. In particular, the distal site of the α chain results to be very unusual since the distal His is displaced from its canonical position. This displacement is coupled with a shortening of the highly conserved E helix and the formation of novel interactions at tertiary structure level. Interestingly, the quaternary structure is closer to the T-deoxy state of Hbs than to the R-state despite the full coordination of all chains. Notably, these peculiar structural features provide a rationale for some spectroscopic properties exhibited by the protein in solution. Finally, this unexpected structural plasticity of the heme distal side has been associated with specific sequence signatures of various Hbs.

## Introduction

The superfamily of globins includes proteins that are ubiquitous in a variety of different living organisms, from bacteria to higher eukaryotes, where they perform key functions essentially related to their ability to bind gaseous ligands^1–3^. The best characterized globins are isolated from vertebrates and include hemoglobin (Hb), myoglobin (Mb), and the more recently discovered cytoglobin (Cygb) and neuroglobin (Ngb). These proteins deserve a special position in structural biology since two members of this family, Mb and Hb, were the first globular proteins whose atomic-level structure was determined. Moreover, comparative analyses of the structural properties of different functional states have greatly contributed to the definition of the well-known paradigm that associates protein function with structure. The globin fold is made of six to eight helices that constitute the matrix where the heme group, which is deputed to the ligand binding, is anchored^3^. Studies on Hbs were also fundamental for understanding protein allostery on structural grounds^4–6^. Although the structure of Hb has been known for approximately sixty years^7,8^, it is still a subject of considerable interest^6,9^. As anticipated above, this is related to the fact that Hb has also been, and still is, one of the prototypical systems for investigating basic structure-function relationships in proteins. Hbs isolated from different sources display a wide range of functional properties that have to be structurally interpreted yet^10^. In this context, the characterization of Hbs isolated from organisms living in extreme conditions has proven to be particularly fruitful. Indeed, studies carried out on Hbs of fish thriving in the freezing water of the Antarctic Ocean have provided interesting insights into the repertoire of structural states that Hbs may assume as well as into the variety of oxidation states that can characterize the heme iron^11–28^. The Southern Ocean, which surrounds Antarctica, is the coldest ocean on Earth and it is isolated from the other oceans by the Antarctic circumpolar current in the last ∼32 million years^29^. Particularly relevant is the observation that structures of Hbs isolated from Antarctic fishes may assume peculiar tertiary and quaternary states that can be defined experimentally at atomic-level^14,15,19^ and are likely only transient in other tetrameric Hbs^30,31^. The characterization of forms that display structures and binding states that are intermediate between canonical R (ligand-bound) and T (deoxy) states provides a unique opportunity to directly visualize these long-sought but elusive functional states that play a key role in the transition. In this scenario, we have undertaken the biochemical and biophysical characterization of the Hb isolated from the sub-Antarctic notothenioid fish *Eleginops maclovinus* (Hb1Em) that lives in the Beagle Channel surrounding the Tierra del Fuego (Argentina) by experiencing temperature ranges from 4 °C to 10 °C^32^. *E. maclovinus* diverged prior to the isolation of Antarctica and is phylogenetically the closest sister species to the modern Antarctic clade. Its genome, recently sequenced, is the best representative of the temperate character of the most recent common ancestor of the Antarctic notothenioids^33^. This species is a close relative of the Antarctic notothenioids such as *Trematomus bernacchii*^12,15,16,23,28,34^ and *Trematomus newnesi*^11,13,19,35^ whose Hbs have been extensively characterized. The spectroscopic characterization of *E. maclovinus* Hb1 has unraveled that the protein presents two distinct CO forms in solution^36,37^. We have previously reported the equilibrium and kinetic study of the oxygenation process for Hb1Em. Moreover, we also investigated the vibrational spectroscopy of the various ferrous and ferric states, along with the crystal structure corresponding to one of these carbomonoxy forms in a canonical R state^36^. Particularly, Hb1Em exhibits the Root effect (drastic drop of oxygen cooperativity at low pH) that is physiologically necessary to secrete O_2_ against high O_2_ pressures into the swimbladder or the retina, following local acidification of the blood in a counter-current capillary system^38^. Hb1Em also presents a biphasic CO dissociation kinetics and multiple CO vibrational frequencies in solution^36^. Here, we report the structure of a second carbomonoxy form that presents several structural properties that are unique for globin structures. These novel crystallographic data also provide some insights into Hb1Em functional properties as well as into the structural basis of some spectroscopic features that are common to the entire Hb superfamily.

## Results

### Binding state and secondary structure

**Figure 1:**
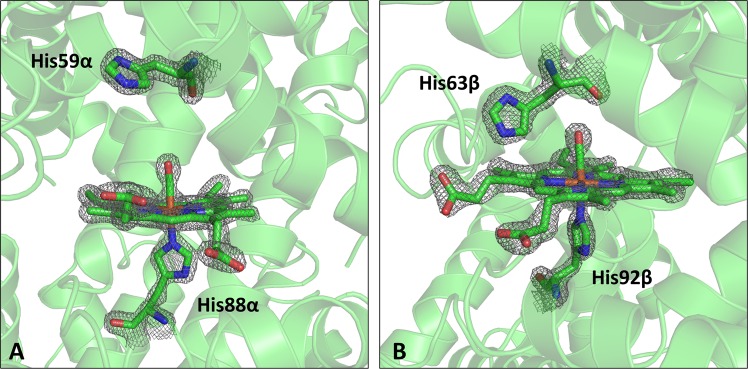
|2Fo-Fc| electron-density maps (contoured at 2.0 σ) of the heme group, proximal and distal His residues and CO molecules involved in the iron coordination of α (**A**) and β (**B**) chains of Hb1EmCO_hexa.

**Figure 2:**
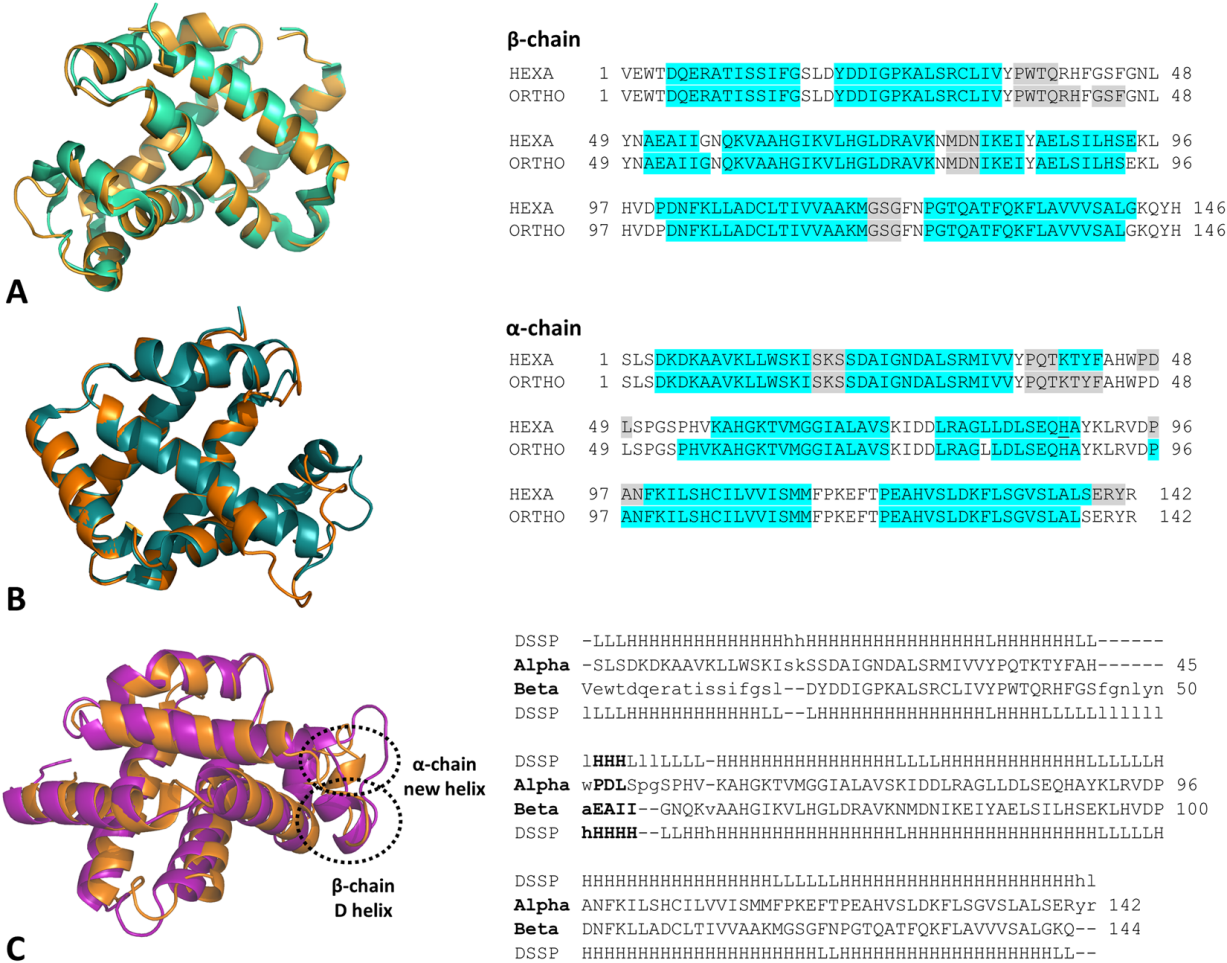
Superimposition of Hb1EmCO_hexa (orange) and Hb1EmCO_ortho (green, PDB ID: 4ESA) β (**A**) and α (**B**) chains. The secondary structure comparison is also reported. The α and 3_10_ helices, assigned using the program DSSP, are highlighted in cyan and grey, respectively. The structural alignment of the α (orange) and β (magenta) chains of Hb1EmCO_hexa is reported in panel C.

The inspection of the electron density at the heme-binding pocket clearly indicates the presence of an exogenous CO molecule bound to the sixth coordination position in both α and β chains of the asymmetric unit (Fig. 1A,B). In both chains, the iron atom is in the plane of the heme group, in line with the general trends observed in hexa-coordinated states. Therefore, the heme binding state of this novel crystal form (hereafter denoted as Hb1EmCO_hexa) is identical to that observed in the carbonmonoxy Hb1Em previously reported (Hb1EmCO_ortho, PDB ID: 4ESA)^36^. Nevertheless, the analysis of Hb1EmCO_hexa indicates that this structure presents a number of striking differences when compared to Hb1EmCO_ortho. It also presents specific features that are rare or unique in the world of tetrameric Hbs. The comparative analysis of the secondary structure elements present in Hb1EmCO_hexa and Hb1EmCO_ortho indicates that the secondary structure of the β chains is rather well-preserved (Fig. 2A). On the other hand, the analysis of Hb1EmCO_hexa secondary structure shows that the α chain of this structure presents significant variations when compared to Hb1EmCO_ortho, although these crystal forms were obtained under the same crystallization conditions^37^. Indeed, as shown in Fig. 2B, the N-terminal end of the E helix (residues 54–56) is unfolded and a small 3_10_ helix is observed for residues 47–49. Notably, this extra helix is in the sequence region that corresponds to the helix D of β chains of globins that is missing in globin α chains (Fig. 2C). The unfolding of the N-terminal region of the α-chain helix E has never been observed in the structures of tetrameric Hbs. Indeed, a survey of the Protein Data Bank (PDB) shows that none of the 1,519 Hb α/β chain structures reported in the PDB (see methods for details), with sequence identities with Hb1Em ranging from 45% to 100%, shows shortening of the helix E in the α chains as detected in Hb1EmCO_hexa.

### Tertiary structure and binding pocket

**Figure 3:**
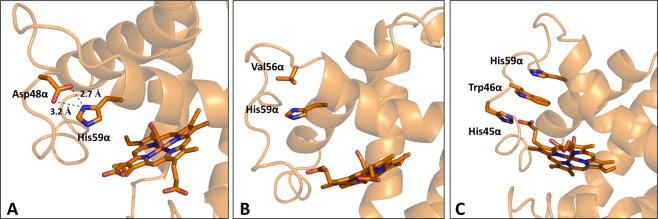
Interactions established by the distal His59 of the α chain in the crystal structure of Hb1EmCO_hexa: electrostatic interaction between His59α and Asp48α side chains (**A**), CH-π interaction between His59α and Val56α (**B**), cluster of stacking interactions involving His45α, Trp46α, and His59α (**C**).

**Figure 4:**
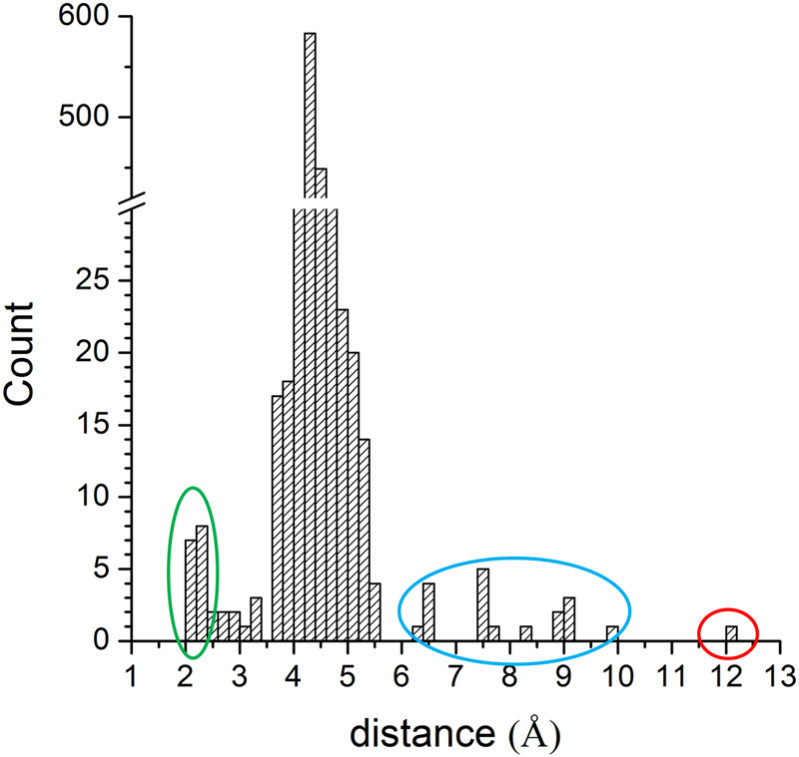
Distribution of distances between the heme iron and the N^ε^ atom of the distal His detected in 1,519 of α/β chains of Hbs reported in the PDB. The ovals highlight the chains in which the distal His is involved in the iron hexa-coordination (green) or the chains in which the His side chain swings out from the heme pocket (blue). The α chain of Hb1EmCO_hexa is highlighted by a red circle.

The structural variations observed in Hb1EmCO_hexa have a remarkable impact on the tertiary structure and on the heme binding pocket of the protein. In the α chain a striking rearrangement of the heme pocket is observed: the side chain of the distal His59α, which assumes a canonical location in Hb1EmCO_ortho, is far from the heme group (Fig. S1A). Indeed, the distance between the N^ε2^ atom of His59α and the oxygen atom of the CO ligand, which are at hydrogen-bonding distance in canonical carbonmonoxy Hbs, is 10.3 Å in Hb1EmCO_hexa (Table S1). This unique location of His59α is favored by the helix E distortion and stabilized by the formation of several stabilizing interactions (Fig. 3). The most evident one is the strong electrostatic interaction established by His59α and Asp48α side chains (Fig. 3A). His59α side chain also interacts with the side chain of Val56α through CH-π interactions (Fig. 3B). Moreover, His59α takes part to a cluster of stacking interactions also involving His45α and Trp46α (Fig. 3C). The displacement of His59α produces a rearrangement of the distal side of the heme pocket characterized by a movement of the side chains of the hydrophobic residues Phe43α, Trp46α, and Leu29α that, compared to Hb1EmCO_ortho, become closer to the CO molecule (Fig. S2). Interestingly, the rearrangement of the overall architecture of the distal side observed in Hb1EmCO_hexa is unique among vertebrate globins. Indeed, the displacement of the His side chain from the heme pocket here observed never occurs in more than 1,500 Hb chains reported in the PDB (Fig. 4) and has never been detected in the structures of other globins such as Mb, Cygb and Ngb (data not shown). Although the overall architecture of the distal side of the β-heme pocket is essentially preserved, it is worth mentioning that, as occasionally observed in other Hbs^11,39^, the distal His63β swings out of the heme pocket (Fig. S1B). Indeed, its χ1 angle (−77.9°) is rather different from that observed in both chains of Hb1EmCO_ortho (ranging from −163° to −156°) which represents the canonical R state CO form. We also evaluated the overall re-organization of the heme pockets by calculating the C^α^-C^α^ distance between the distal and proximal His residues. This analysis provides further support to the unusual structural properties of Hb1EmCO_hexa as, in both chains, the distances of the C^α^ atoms between the distal and proximal histidines in this structure are quite different from those observed in the canonical R state (Fig. S1). The higher value observed for the α chain (15.8 Å in Hb1EmCO_hexa *versus* 14.3 Å in Hb1EmCO_ortho) is clearly due to the displacement of the distal site (Table S1). On the other hand, the lower value detected for the β chain (12.8 Å in Hb1EmCO_hexa *versus* 14.1 Å in Hb1EmCO_ortho) is due to compression of the EF corner associated with the swinging out of the side chain of the distal histidine (Table S1).

### Quaternary structure

**Figure 5:**
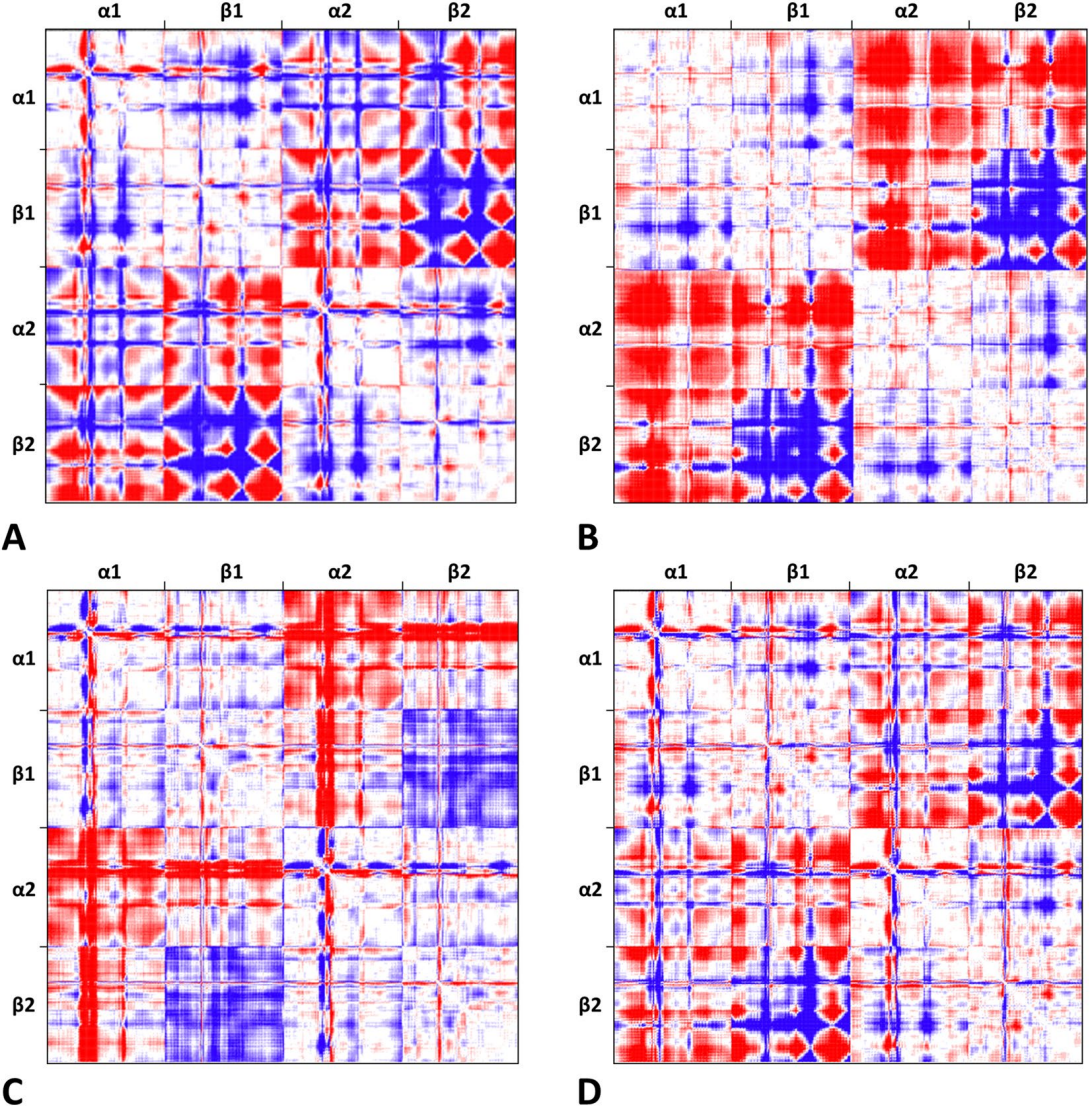
Localization of the quaternary state of Hb1EmCO_hexa tetramer along the R-T pathway. Difference-distance matrices of Hb1EmCO_hexa *versus* Hb1EmCO_ortho (PDB ID: 4ESA) (**A**), Hb1Tn deoxy (PDB ID: 3NFE) *versus* Hb1TnCO (PDB ID: 1T1N) (**B**), Hb1Tn deoxy *versus* Hb1EmCO_hexa (**C**), and Hb1EmCO_hexa *versus* Hb1TnCO (**D**). The color range extends from -2 Å (red) to 2 Å (blue).

**Figure 6:**
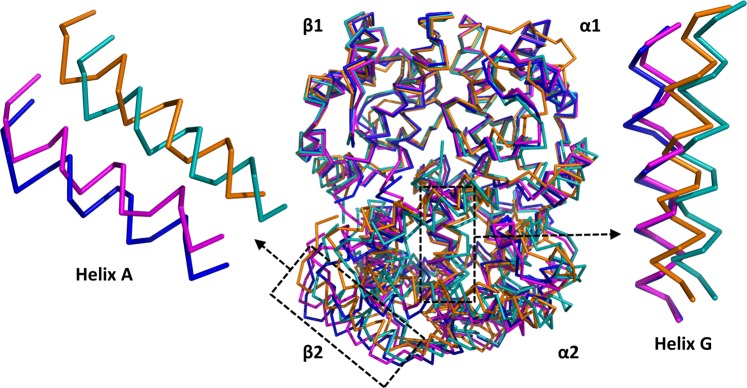
Superimposition of the α1β1 dimer of Hb1EmCO_hexa (orange), Hb1EmCO_ortho (blue, PDB ID: 4ESA), Hb1Tn deoxy (cyan, PDB ID: 3NFE), and Hb1TnCO (magenta, PDB ID: 1T1N). The helices A (residue 3–18) and G (residues 99–117) of the β2 chain are shown to highlight the transition from the R to the T state.

To evaluate the impact on Hb1EmCO_hexa quaternary structure of the observed structural peculiarities at secondary and tertiary structure levels we applied a number of global and local indicators. These analyses were specifically designed to locate this non-canonical structure in the framework of the R-T transition characteristic of tetrameric Hbs. Taking into account the close structural analogy of Hb1EmCO_ortho with canonical CO-bound forms of tetrameric Hbs, Hb1EmCO_ortho was used as the standard R-state of Hb1Em. On the other hand, the lack of structural characterizations of Hb1Em in its T state prompted us to use as a framework for the R-T transition the R (Hb1TnCO)^35^ and the T (Hb1Tn deoxy)^40^ states of the closely related Hb1 from *T. newnesi* (Hb1Tn). Pairwise comparisons of these structures were initially performed by considering root mean square deviation (RMSD) values, computed on the C^α^ atoms of the isolated chains and the dimer/tetramer assembly (Table 1). The inspection of the RMSD values shows that the quaternary structure of Hb1EmCO_hexa is quite different from that exhibited by Hb1EmCO_ortho (RMSD of 2.16 Å). Moreover, it is somehow intermediate between the R and the T state of Hb1Tn. A deeper analysis of the RMSD values indicates that Hb1EmCO_hexa is closer to Hb1Tn T state (RMSD of 1.18 Å) than to the R state (RMSD of 1.73 Å). To better localize the structure of Hb1EmCO_hexa in the R-T pathway we calculated difference distance matrices (DDMs). In line with the findings described above, the DDM obtained from the comparison of Hb1EmCO_hexa and Hb1EmCO_ortho clearly indicates that Hb1EmCO_hexa structure is rather different from the canonical R state, despite the identical binding state of all iron atoms (hexa-coordination with a CO molecule). Interestingly, the difference matrix between Hb1EmCO_hexa and Hb1EmCO_ortho (Fig. 5A) resembles the one computed between the T and R states of Hb1Tn (Fig. 5B). Indeed, the inspection of these matrices indicates that the differences in the juxtaposition of the α1/β2 and β1/β2 subunits are rather similar in Hb1EmCO_ortho/Hb1EmCO_hexa and in Hb1TnCO/Hb1TnT. On the other hand, the relative orientation of α1/α2 subunits is different in these two systems. The visual inspection of these DDM matrices also suggests that Hb1EmCO_hexa is closer to Hb1TnT than Hb1TnCO (Fig. 5C,D). This is line with the RMSD values obtained from the pair-wise comparisons (Table 1). Collectively, the analysis of the difference matrices generated by comparing Hb1EmCO_hexa *versus* the T state and the R state of Hb1Tn (Fig. 5C,D) confirms that this novel structure is shifted toward the T state in the R-T pathway, as also showed by the superimposition of these Hb structures reported in Fig. 6. The evidence in Hb1EmCO_hexa of a stable carbomonoxy form in a T quaternary state is in perfect agreement with a biphasic CO-dissociation kinetic and with the stopped-flow kinetic curves previously observed for the Hb of *E. maclovinus*^36^. The monitoring of specific structural features that differentiate T and R states of Hbs corroborates and strengths the picture emerging from the global analysis of Hb1EmCO_hexa quaternary structure. One of the key points that characterize the R-T transition is the relative position of His97β2 of the β2 FG corner and helix C of the α1 chain (Fig. S3). In Hb1EmCO_hexa, the location of the histidine is intermediate between that observed in T and R states. The T states of fish Hbs are frequently characterized by the formation of a peculiar interaction between the side chains of Asp95α1 and Asp101β2 that is stabilized by Asp99β2^16,17,41,42^. Although this carboxyl-carboxylate interaction is not observed in Hb1EmCO_hexa, these aspartic acid residues are closer in this structure when compared to Hb1EmCO_ortho (Fig. S4). It should be noted that Hb1EmCO_hexa crystals were obtained at a pH value (7.6) that does not favor this type of interaction and may contribute to impede the complete R-T transition of Hb1EmCO_hexa. Finally, Hb1EmCO_hexa also lacks the salt bridge formed by Lys40α1 side chain and the C-terminal carboxyl group of His146β2, which is considered a fingerprint of the T state in vertebrate Hbs, due to structural disorder observed for residues 145–146 of the β chain.

**Table 1:** Pair-wise comparisons in terms of RMSD values (Å) computed on the C^α^ atoms between Hb1EmCO_hexa and other Hbs from *E.*

Hb1EmCO_hexa	Hb1EmCO_ortho (4ESA)	Hb1Tn deoxy (3NFE)	Hb1TnCO (1T1N)
α chain	0.70	0.68	0.57
β chain	0.63	0.68	0.74
α_1_β_1_ dimer	0.89	0.77	0.88
tetramer	2.16	1.18	1.73

*maclovinus* (Hb1EmCO_ortho) and *T. newnesi* (T and R states). PDB codes are reported in brackets.

## Discussion

The carbonmonoxy structure of Hb1EmCO_hexa here described presents a number of features that are unique in the universe of globins. These features are not artifact of the crystalline state since vibrational spectroscopy solution studies have shown that two distinct CO forms of Hb1EmCO coexist in solution^36^. Vibrational spectra provide a direct way to estimate the relative amount of these two CO conformers in solution. An abundance of roughly 95% and 5% is estimated for the band at 1954 and 1970 cm^−1^, respectively. The most abundant form, which presents rather standard spectroscopic features (band at 1954 cm^−1^), is likely associated with previously described typical canonical R states, with CO that is hydrogen bonded to the distal histidine. The other form detected in solution, which presents a different higher vibration frequency (band at 1970 cm^−1^), could correspond to the non-canonical model here described where CO does not interact with the distal histidine, which swings out from the heme pocket. In support to this interpretation, there are several experimental and theoretical studies. In particular, an extra band at 1970 cm^−1^ has been previously observed in Hbs of temperate (carp)^43^, sub-Antarctic^36^, and Antarctic^11^ fish, and of some rodents^43^. It has also been reported for wild-type HbA under denaturing conditions^43^, in phosphate-buffered solutions, and, in low amounts, even in red blood cells^44^. More interestingly, the CO stretching band around 1970 cm^−1^ was observed for several distal HbA mutants such as Tyr63βHis Emory^45^ and Arg63βHis Zurich^46^. Finally, in support to our structural interpretation of the two CO vibrational bands at 1954 and 1970 cm^−1^, a direct correspondence between CO coordination and band assignment is also in line with previous experimental theoretical studies on the occurrence of the 1970 cm^−1^ band in hemoproteins^47–50^. Indeed, according to these theoretical studies, the upshift in the CO stretching can well be assigned to the loss of the H-bond between N^ε^ of the distal His imidazole ring and CO that leads to a more hydrophobic environment surrounding the CO ligand.

This structural assignment of the two vibrational bands at 1954 and 1970 cm^−1^ with a closed or an open local environment of the CO ligand perfectly fits with the displacement of the distal histidine from its canonical position and the consequent rearrangement of the local architecture of the distal side. It is important to note that this rearrangement involves a significant reorganization of the residues 43–48 of the α chain. This region embodies the fish-specific insertion in position 47 that makes the α chain of fish Hb one residue longer (Fig. S5). The presence of an additional residue in this region is important for the reorganization here observed. These considerations may explain the higher tendency of fish Hbs to exhibit the band around 1970 cm^−1^ in the FT-IR spectrum. Interestingly, an enhanced band around 1970 cm^−1^ is also displayed by rat Hb compared to human HbA^43^. Although the rat sequence, like all mammalian Hbs, does not contain extra residues in the 43–48 region of the α chain, its local sequence is significantly different from HbA. It is also worth to remind that relevant modifications in the CDα region in mammalian Hbs are tightly associated to the EFα contraction observed in bis-histidyl complex formation, crystallographically observed at acidic pH for horse Hbs^31^. Indeed, the sequence alignment reveals that, in rat Hb, Pro45 of HbA is replaced by a Ser residue, which is more flexible (Fig. S5). Moreover, in the human sequence His51 establishes a strong electrostatic interaction with Glu31 side chain that is absent in the rat Hb since His51 is replaced with a Pro residue. It is likely that these mutations endow the rat Hb with an increased flexibility of the 43–48 region that generates an enhanced band around 1970 cm^−1^ in the FT-IR and Resonance Raman spectrum. It is also noteworthy that the unusual alterations of the secondary and tertiary structures of the protein leads to a quaternary structure that still falls in the R-T functional transition of tetrameric Hbs. These findings suggest that although proteins may be endowed with local structural versatility, they likely possess a limited number of possible overall structural transitions.

Besides the general implications on the possible CO conformers and on the R-T transitions, our study offers a structural interpretation for specific properties of Hb1Em. The observed biphasic CO dissociation^36^ completely matches with the observation both in solution and in the crystal structures of two distinct CO conformers that are expected to present drastically different CO-dissociation rate constants. Moreover, the value of the faster k_off_ for Hb1Em reveals a weak Fe-CO bond. This is clearly anticorrelated to the strong CO stretching at 1970 cm^−1^ due to the higher extent of π-back-bonding. Moreover, under the hypothesis that the two kinetic phases could be associated with the two CO conformers, the biphasic CO dissociation curves imply a quite slow interconversion rate between the two conformers. The slow interconversion, which implies a high free energy barrier, may well be related to the large marked changes observed between the previous R^36^ and the current crystal structure of Hb1Em.

As observed for Hb1Em, autoxidation is very fast in fish and in particular in Antarctic and sub- fish Hbs^13,18^. This evidence can well be related to the presence in most of fish Hbs of significant amounts of the conformer(s) in which the CO ligand is not bound to the distal histidine (band around 1970 cm^−1^).

In conclusion, after sixty years of reports, structural characterization of Hb structures may still unravel unexpected findings. The structural characterization of Hb1EmCO here described shows an unexpected plasticity at the heme distal side, a key functional region that was believed to be strictly structured in tetrameric Hbs.

## Materials and Methods

### Protein purification, crystallization and data collection

Specimens of *E. maclovinus* were collected during the ICEFISH 2004 cruise close to the Falkland Islands. Blood samples were taken from the caudal vein. Separation of *E. maclovinus* hemoglobins was accomplished by FPLC anion exchange chromatography using a Mono Q-Tricorn column. Hb1Em was further purified by ion-exchange chromatography (see ref. ^37^ for further details about protein purification procedures).

A full description of the crystallization and data collection of Hb1Em in the carbonmonoxy form has been previously reported in detail^37^. Briefly, the protein was crystallized at 277 K in a CO atmosphere using the dialysis technique with microdialysis buttons. Na_2_S_2_O_4_ was added to the crystallization solution to guarantee a reducing environment hampering autoxidation. The best crystals of Hb1Em were obtained using a protein concentration of 20 mg ml^−1^ and 1.8 M ammonium sulfate pH 8.0. Diffraction data were collected at the ELETTRA synchrotron and in-house using a Rigaku MicroMax-007 HF generator equipped with a Saturn944 CCD detector. Cryoprotection of the Hb1Em crystals for diffraction at 100 K was achieved by adding 20% glycerol and Na_2_S_2_O_4_ to keep the ferrous state to the harvesting solution. The data sets were scaled and merged using the HKL2000 program package^51^. The crystal structure of Hb1Em_hexa was solved by molecular replacement using the program Phaser^52^ and the structure of the αβ dimer of Hb1EmCO belonged to the space group P2_1_2_1_2_1_ (PDB ID: 4ESA) as starting model. Then, an automatic rebuilding was performed using ARP/wARP^53^. Crystallographic refinement was carried out using 95% of the measured data with the ccp4i program. In order to monitor the progress of refinement, the remaining 5% of the observed data, which was randomly selected, was used to calculate the Rfree. The program REFMAC was used for the refinement^54^. The refinement details along with the statistics of the final protein models are given in Tables 2 and 3. As the two models are virtually identical, all of the analyses and descriptions reported in the text refer to the structure derived using the highest resolution synchrotron data. The stereochemistry of Hb1EmCO_hexa has been evaluated by using both standard protocols such as PROCHECK^55^ and innovative approaches based on the monitoring of fine details of the protein backbone geometry^56–58^. In this latter approach, we evaluated the variability as function of local conformation of geometrical parameters like bond angles, the peptide bond planarity, and the carbon carbonyl pyramidalization and compared it to that observed in high resolution and well refined protein structures (Fig. S6 and Table S2) (Vitagliano *et al*. in preparation) (see Supplementary Material for the details). Atomic coordinates of this model have been deposited in the PDB with the identification code 6RP5.

**Table 2:** X-ray data collection statistics of Hb1EmCO_hexa.

X-ray device	ELETTRA synchrotron	Rigaku FR007HF with CCD detector
Space group	P 6_1_ 2 2	P 6_1_ 2 2
**Unit-cell parameters**		
a, b, c (Å)	91.70, 91.70, 168.72	91.63, 91.63, 169.12
α, β, γ (°)	90.0, 90.0, 120.0	90.0, 90.0, 120.0
Asymmetric unit	αβ dimer	αβ dimer
**Data processing**		
Resolution limits (Å)	79.4–1.49	79.4–2.03
No. of reflections	212199	86031
No. of unique reflections	66710	25051
Completeness (%)	96.4 (92.5)	89.6 (65.2)
I/σ (I)	8.0 (2.1)	24.7 (3.0)
Average multiplicity	3.2 (2.6)	3.4 (2.6)
Rmerge	0.095 (0.440)	0.071 (0.329)

Values in brackets are for the highest resolution shell (1.53–1.49 Å and 2.03–2.10 Å for Hb1EmCO_hexa X-ray data collected at ELETTRA synchrotron and in-house, respectively).

**Table 3:** Refinement statistics of Hb1EmCO_hexa.

X-ray device	ELETTRA synchrotron	Rigaku FR007HF with CCD detector
R/Rfree (%)	16.8/19.6	18.0/22.6
No. of non-hydrogen atoms	2765	2564
No. of water molecules	389	229
No. of protein residues	286	286
**RMSD from ideal values**		
Bond lengths (Å)	0.022	0.017
Bond angles (°)	2.14	1.83
Mean B-factor (Å^2^)	34.6	38.2
**Ramachandran plot statistics of Procheck**		
Residues in most favoured regions	239	239
Residues in additional allowed regions	14	14

### Statistical surveys of Hbs structures

Three-dimensional structures of Hbs have been selected from the PDB by using BLAST (https://web.expasy.org/blast/). The statistical analyses of the heme pocket on the resulting 1,519 structures have been performed by computing the distance between the heme iron and the N^ε^ atom of the distal His in both α and β chains.

## Supplementary information

Supplementary info
