# Supplementary material for: The unique structural features of carbonmonoxy hemoglobin from the sub-Antarctic fish Eleginops maclovinus
Source: Sci Rep. 2019 Dec 12;9:18987. doi: 10.1038/s41598-019-55331-3 (PMC6908587; doi:10.1038/s41598-019-55331-3)
Supplement: Supplementary file 1 — Supplementary info [file 41598_2019_55331_MOESM1_ESM.docx]

**Supplementary Material**

The unique structural features of carbonmonoxy hemoglobin from the sub-Antarctic fish *Eleginops maclovinus*

Nicole Balasco^1^, Luigi Vitagliano^1*^, Antonello Merlino^2^, Cinzia Verde^3^, Lelio Mazzarella^2^, and Alessandro Vergara^2*^

^1^Institute of Biostructures and Bioimaging, CNR, Via Mezzocannone 16, Naples, Italy

^2^Dept. Chemical Sciences, University of Napoli “Federico II” Via Cinthia, 80126, Naples, Italy

^3^Institute of Biosciences and BioResources, CNR, Via Pietro Castellino 111, 80131, Naples, Italy

**
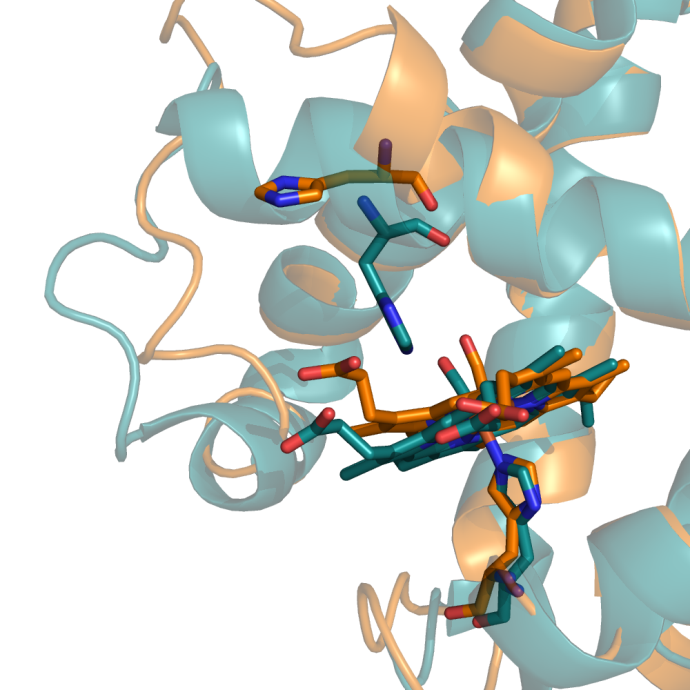

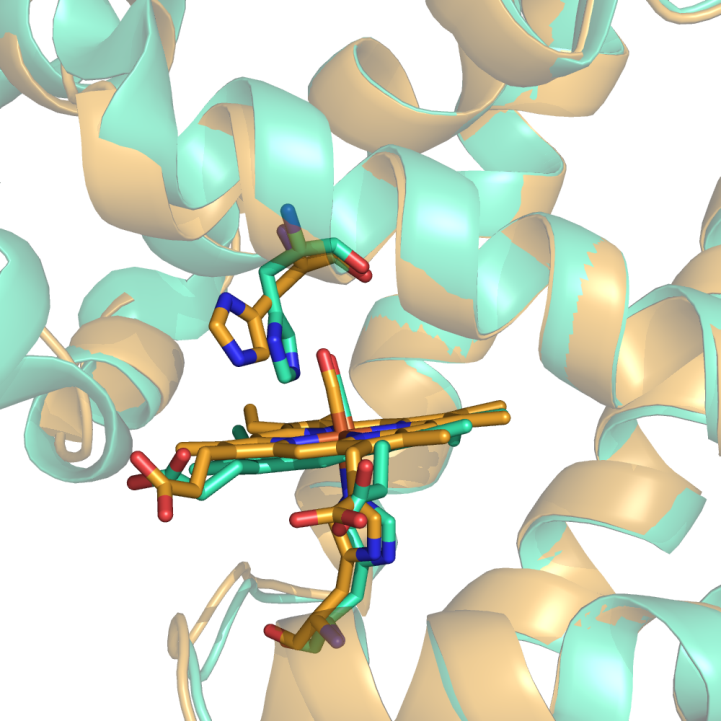
**

**His92β**

**His63β**

**His59α**

**His88α**

**A B**

**Figure S1.** Superimposition of the binding pocket region of Hb1EmCO_hexa (orange) and Hb1EmCO_ortho (green, PDB ID: 4ESA). The α and β chains are shown in panel A and B, respectively.


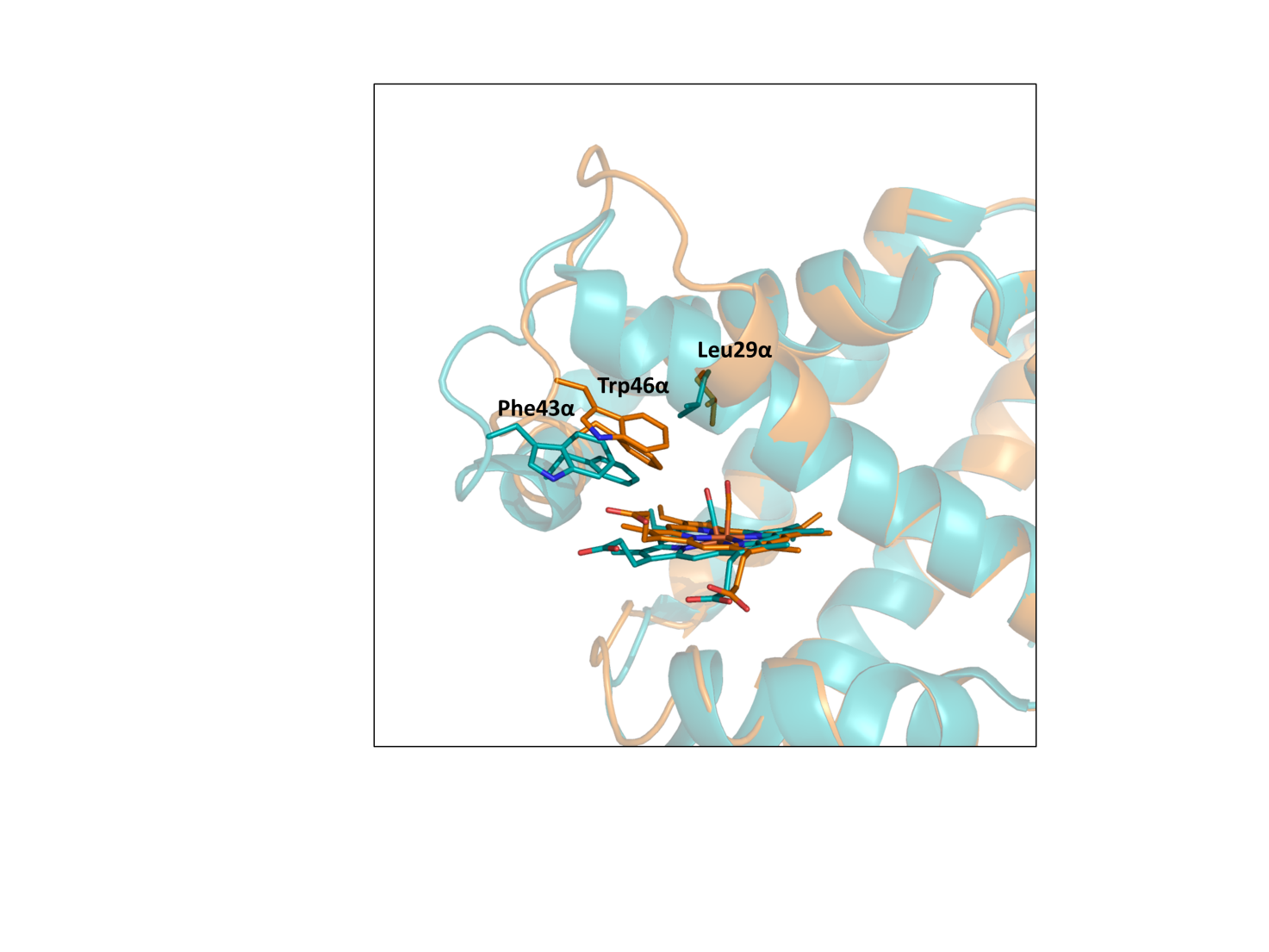


**Figure S2.** Superimposition of the binding pocket region of Hb1EmCO_hexa (orange) and Hb1EmCO_ortho (green, PDB ID: 4ESA). The rearrangement of the distal side of the heme pocket characterized by a movement of the side chains of the hydrophobic residues Phe43α, Trp46α, and Leu29α that in Hb1EmCO_hexa, compared to Hb1EmCO_ortho, become closer to the CO molecule is shown.


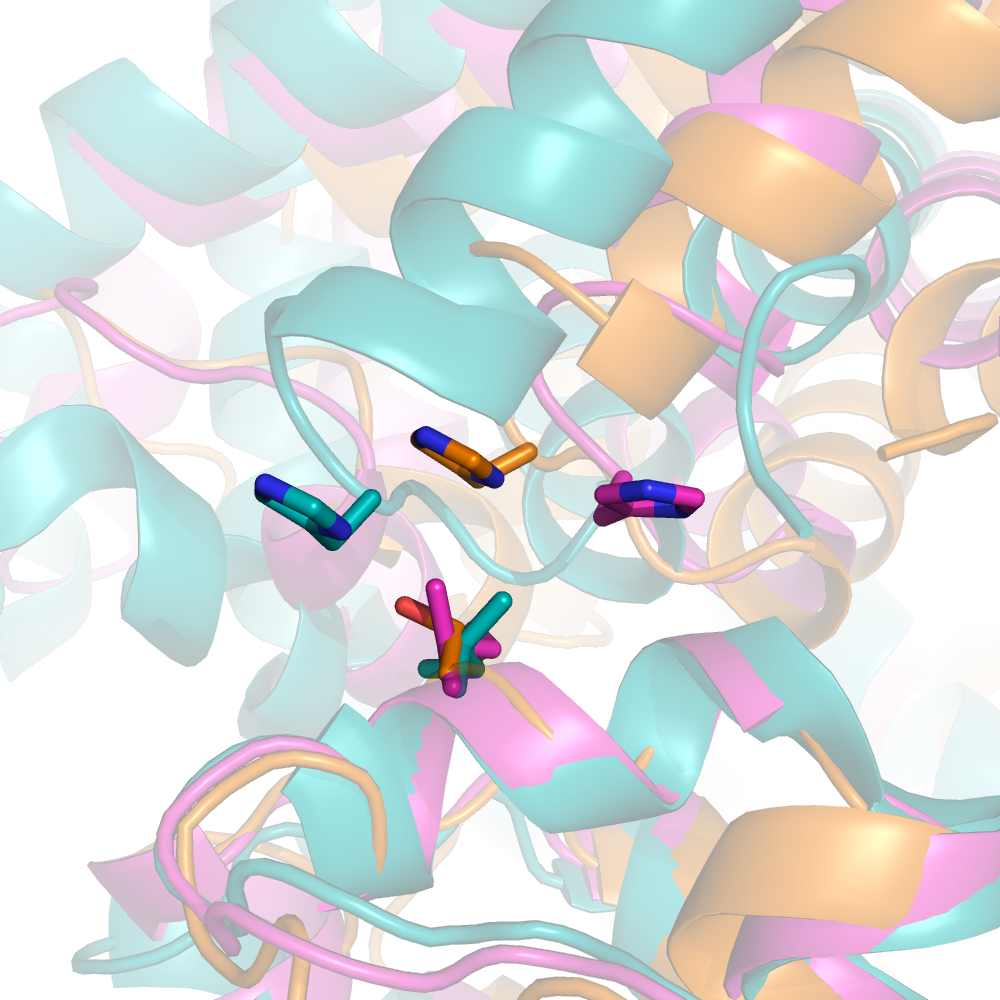


**His97β2**

**Ile41(Thr41)α1**

**Figure S3.** Superimposition of the switching region composed by helix C of the α1 chain and the β2 FG corner: Hb1EmCO_hexa (orange), Hb1TnT (green, PDB ID: 3NFE), Hb1TnCO (magenta, PDB ID: 1T1N). Residues Ile41α1 (or Thr41α1) and His97β2 are shown.


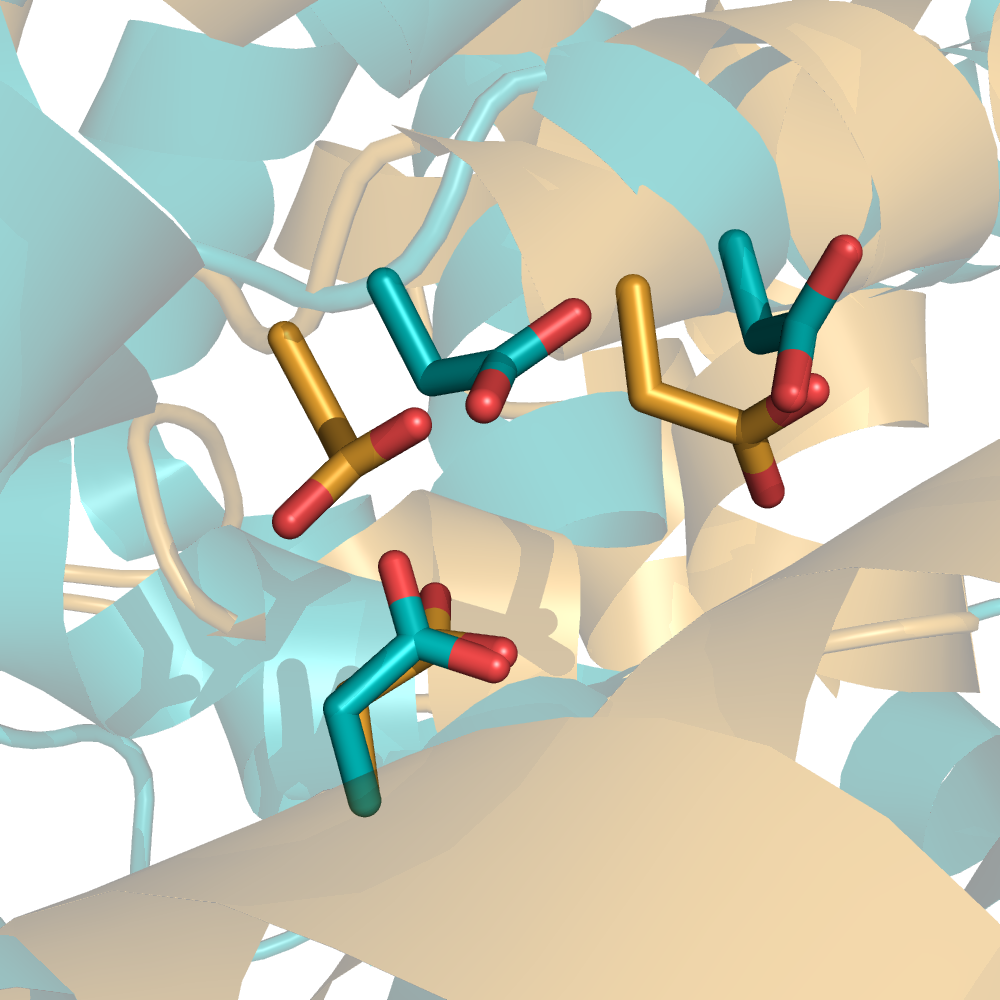


**Asp101β2**

**Asp99β2**

**Asp95α1**

**Figure S4.** Interaction between the side chains of residues Asp95α1, Asp99β2, and Asp101β2 at the α1β2 interface of Hb1EmCO_hexa (orange) and Hb1Em_ortho (green, PDB ID: 4ESA).

HBA1_ELEMC 35 VYPQTKTYFAHWPDLSPGSPH 55

HBA1_TRENE 35 VYPQTKIYFSHWPDVTPGSPN 55

HBA_PSEBE 35 VYPQTKTYFSHWPDVTPGSPH 55

HBA_RAT 36 AFPTTKTYFSHI-DVSPGSAQ 55

HBA_HUMAN 36 SFPTTKTYFPHF-DLSHGSAQ 55

**Figure S5.** Sequence alignment of the region 35-45 of the α chain of Hb sequences from different species (*Eleginops maclovinus, Trematomus newnesi, Pseudotrematomus bernacchii, Rattus norvegicus and Homo sapiens*). The most significant differences between rat and human sequences are highlighted in yellow.


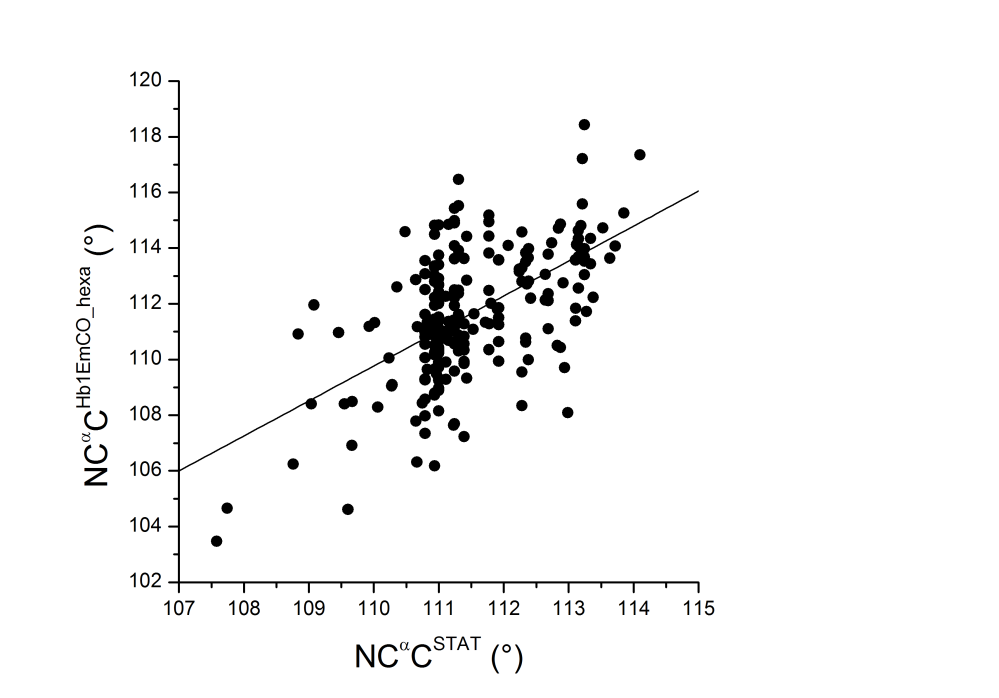

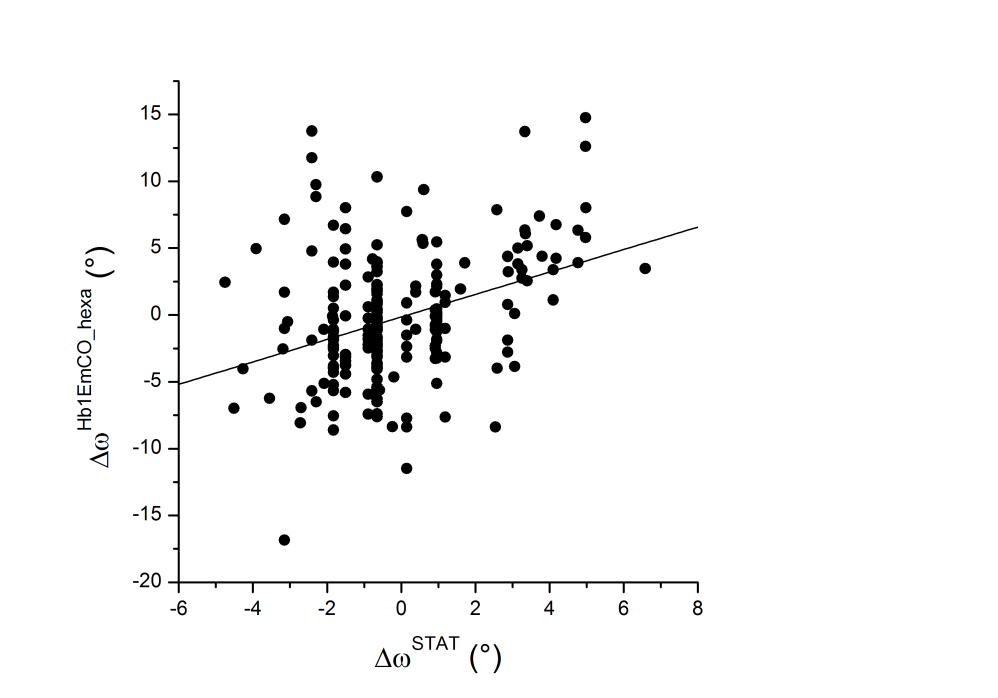


**A B**

**Figure S6.** Validation of the variability of some geometrical parameters of the protein backbone: the NC^α^C bond angle (A) and the deviation from the peptide bond planarity Δω=(ω-180°)mod360° (B) of Hb1EmCO_hexa crystal structure. The regression analysis has been performed by plotting the NC^α^C or Δω values of Hb1EmCO_hexa residues versus the average NC^α^C or Δω values of residues adopting the same (φ, ψ) conformation obtained from databases of X-ray PDB structures solved at high resolution (release of March 2016). These protein structures were selected using the PISCES culling server (http://dunbrack.fccc.edu/PISCES.php) applying the following criteria: resolution better than 1.6 Å for bond angles and 1.2 Å for dihedral angles, R-factor ≤ 0.20, and sequence identity ≤ 25%. Additional selections were carried out at residue level by excluding residues for which the ratio between the average backbone B-factor (atomic displacement parameter) of the residue and the same parameter computed considering the entire chain was higher than 1.3. These databases contain 3,291 (bond angles) and 799 (dihedral angles) non-redundant protein chains. The regression lines are shown. The parameters (correlation coefficient and p-value) of the linear fitting are reported in Table S2.

**Table S1.** Heme stereochemistry of Hb1EmCO_hexa and Hb1EmCO_ortho (PDB ID: 4ESA). Distances are reported in Å.

| **Distance** | **Hb1EmCO_hexa** | | **Hb1EmCO_ortho** | |
| --- | --- | --- | --- | --- |
|  | ***α chain*** | ***β chain*** | ***α chain*** | ***β chain*** |
| **CO-N^ε2^ (HisE7)** | 10.3 | 5.5 | 3.23 | 3.32 |
| **C^α^(HisF8)-C^α^(HisE7)** | 15.8 | 12.8 | 14.3 | 14.1 |

**Table S2.** Results of the regression analysis of the variability of some geometrical parameters (bond angles, deviation from peptide bond planarity Δω and carbon carbonyl pyramidalization θ_C_) of Hb1EmCO_hexa structure versus datasets of parameters derived from well-refined structures (see text and legend of Figure S6). This regression analysis indicates that the variability of geometrical parameters well follows that detected in well refined protein structures.

| **Geometrical parameter** | **Correlation coefficient** | **p-value** |
| --- | --- | --- |
| NC^α^C | 0.57 | <10^-10^ |
| NC^α^C^β^ | 0.11 | 0.088 |
| C^β^C^α^C | 0.28 | 1.4*10^-5^ |
| C^α^CO | 0.37 | <10^-10^ |
| C^α^CN_+1_ | 0.30 | 5.0*10^-6^ |
| OCN_+1_ | -0.012 | 0.86 |
| C_-1_NC^α^ | 0.49 | <10^-10^ |
| Δω | 0.35 | <10^-10^ |
| θ_C_ | 0.23 | 5.6*10^-4^ |
